# Maintenance of occupational therapy (OT) for dementia: protocol of a multi-center, randomized controlled and pragmatic trial

**DOI:** 10.1186/s12877-019-1046-x

**Published:** 2019-02-06

**Authors:** Clément Pimouguet, Rémi Sitta, Jérôme Wittwer, Nathalie Hayes, Aurélie Petit-Monéger, Jean-François Dartigues, Catherine Helmer

**Affiliations:** 1 Bordeaux Population Health Research Center, University Bordeaux, Inserm, ISPED, 146 rue Léo Saignat, CS61292, F-33076 BORDEAUX Cedex, UMR 1219, F-33000 Bordeaux, France; 2 Methodology Research Unit, Bordeaux University Hospital, Bordeaux, France; 3 Direction de la recherche clinique et de l’innovation, evaluation et accompagnement de l’innovation, Pole de sante publique, Service d’Information Medicale, CHU de Bordeaux, 33000 Bordeaux, France; 4 CHU de Bordeaux, place Amelie Raba-Léon, 33000 Bordeaux, France; 5 Memory Consultation, CMRR, Bordeaux University Hospital, F-33076 Bordeaux, France; 6 Clinical Investigation Center – Clinical Epidemiology 1401, INSERM, Bordeaux, France

**Keywords:** Alzheimer’s disease, Dementia, Occupational therapy, Randomized controlled trial, Informal caregiver, Neuropsychiatric symptoms, Home intervention

## Abstract

**Background:**

There is a growing interest in developing tailored non-pharmacological strategies to face patients’ needs in dementia. Occupational therapy (OT) may contribute to promote self-empowerment of both patients and caregivers. France has implemented nationwide OT over a short-term period of 3/4 months. The main objective of the MathéoAlz study is to measure the impact of maintaining OT over 4 supplementary months on patients’ neuropsychiatric symptoms.

**Methods/design:**

The MatheoAlz trial (Maintenance of Occupational Therapy in Alzheimer’s disease) is a multi-center, pragmatic randomized controlled trial testing maintenance of OT over 4 supplementary months compared to routine OT delivered as recommended. This paper describes the study protocol. MatheoAlz plans to enroll 240 dyads, i.e. dementia patients and caregivers, whose main inclusion criteria are: prescription for routine OT, patients with mild or moderate dementia, living at home, receiving support from an informal caregiver. The study will compare a control group of patients benefiting from 12 to 15 initial sessions of OT over 3/4 months and an intervention group of patients benefiting from these initial sessions plus 8 extra home sessions over 4 supplementary months. The main outcome is the patient’s neuropsychiatric symptoms assessed by the Neuropsychiatric Inventory at 8 months. Several clinical outcomes and economic consequences are measured at 4, 8 and 12 months.

**Discussion:**

This is the first trial designed to assess the specific impact of the maintaining OT on the patients’ neuropsychiatric symptoms burden. The results will inform policymakers on strategies to implement in the near future.

**Trial registration:**

This trial was registered at ClinicalTrials.gov on February 16, 2018, identifier: NCT03435705.

## Background

About 6 million people are currently affected by dementia in Europe [1]. The majority of people with dementia live at home. Living in their own familiar environment may enable people with dementia to maintain their social networks and enjoy a better quality of life [2]. However, they experience a progressive cognitive and functional decline, limiting their ability to perform activities and to communicate; they also have frequent behavioral symptoms that are challenging for the caregivers and interfere with their daily functioning [3]. The progressive loss of social interactions contributes to reduce personal engagement in meaningful daily activities [4, 5]. Yet, engaging persons with dementia in personally-tailored activities may have positive effects on their challenging behaviors and quality of life. These benefits might positively influence caregivers’ burden and well-being. In France, a massive effort was made to optimize dementia care through a national Alzheimer plan 2008–2012 [6]. Several innovative health care services have been implemented nationwide, notably home services offering care plan based on occupational therapy (OT) model. OT allows tailored home support by cognitive and social rehabilitation for subjects with mild-to-moderate dementia; it aims to restore or mobilize the remaining abilities of individuals and adapt their domestic environment. The French OT model involves occupational therapists, psychomotor therapists and gerontologic assistants.

A growing interest in OT currently exists and some trials are ongoing [7, 8]. A review has concluded that literature provided a “proof of concept” that functional decline related to dementia may be delayed via OT interventions [9]. A 5-weeks OT’s program demonstrated clinical efficacy on daily functioning at 12 weeks in the Netherlands [10–12]. Nevertheless, the intervention’s replication in another national context failed to demonstrate any effectiveness [13]. Moreover, a recent US trial failed to demonstrate that OT added to collaborative care improves daily functioning over a 2-year period. It remains unclear whether results observed in efficacy trials with convenience and homogeneous samples are similarly obtained in routine care context. Participants who are typically volunteers for such trials differ from non-volunteers providing similar levels of care [14]. Yet, real-world studies are scarce. Recently, within an observational study based on routine care practice we showed that patients benefiting from OT experienced a stabilization in their functional performances over the 3/4 months intervention period before a worsening phase [15]. They also reported a decrease of their behavioral troubles followed by a stabilization phase. The French model of OT is a short-term intervention that enters into the intimacy of the daily functioning of patients and their caregivers, and the end of the intervention can be disruptive in a context of social and psychological frail equilibrium. Moreover, planning and implementation of relays (such as speech therapists, physiotherapists or personal care assistant) after OT are crucial to maintain therapeutic approaches according to patients’ care needs. Nevertheless, these tasks are time consuming and complex to accomplish in the devoted period. This report describes a protocol being used in an ongoing randomized trial aiming to provide evidence on long-term clinical effectiveness and economic consequences of maintaining OT over 4 extra months.

## Methods/design

### Aims and study hypothesis

The MatheoAlz trial (Maintenance of Occupational Therapy in Alzheimer’s disease) is a multi-center, pragmatic randomized controlled trial testing routine OT delivered as recommended compared to maintenance of OT over four supplementary months. After completion of the initial clinical assessment, participants are randomly allocated into the control group (treatment as usual with 12 to 15 home sessions over 3/4 months) or the treatment group (12 to 15 home sessions over 3/4 months + extra 8 sessions over 4 months). In order not to influence care practice and care organization in the control group, we decided to adopt a pragmatic approach. Thus, ESAs therapists provide routine care as usual in patients randomized in the control group. In this way, maintenance of OT will be compared to real-world care provided by ESAs therapists.

### Setting and participants

The study is being conducted in the South West of France; it began in 2018 and will run until 2019. Recruitment takes place through ESA (French acronym for “Equipe Spécialisée Alzheimer”) that are specialized teams of health professionals intervening at home for dementia patients. Patients are referred to ESA under medical prescription. We selected 10 ESA that were already involved in the pilot study (described elsewhere) and showed a high recruitment capacity [15]. Moreover, patients cared by these settings have been shown to be representative of the patients care by ESA at the national level [16]. All ESA staff met University research team for research compliance and human subject data collection. Eligibility criteria were pragmatically chosen to be representative of situations encountered by therapists in their routine care practice. Patients’ inclusion criteria include having a dementia diagnosis, being referred to an ESA for the first time, a MMSE score over 15, living in the community or in residential care setting and having a non-professional caregiver. Exclusion criteria are listed as follow: institutionalized patients or patients with a short-term project of institutionalization, patients with reported behavior of care refusal, patients already included in a non-pharmacological trial, patients under legal protective measures or not able to provide their written consent for participation in the study, patients or caregivers with characteristic that could affect participation (e.g.: major physical illness; sensory impairment; disability). People with dementia meeting the inclusion criteria will be recruited over an inclusion period of 10 months, this timeframe was estimated based on ESA’s inclusion capacity.

### Study procedures and randomization

**Fig. 1:**
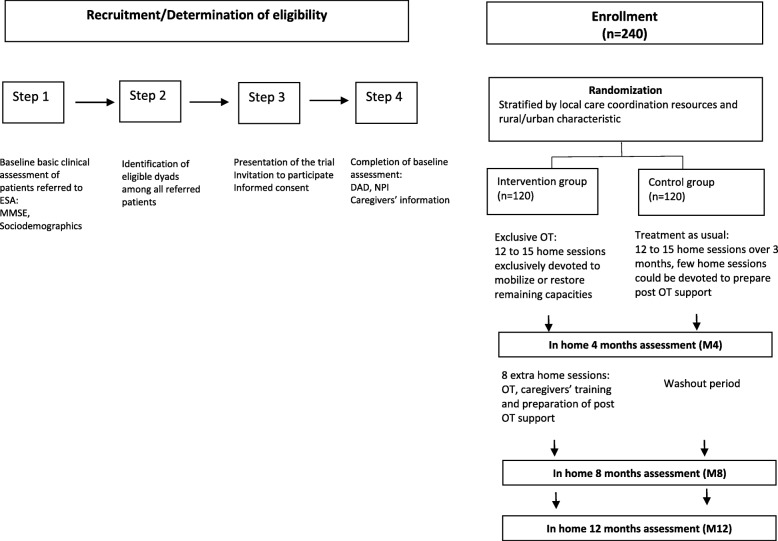
Flowchart of study design

Randomization will be performed with a 1:1 ratio, and will be stratified by territory nature (urban vs rural) and presence of gerontological coordination center in the area (yes vs no) in order to avoid imbalances on major characteristics that might influence care strategies and care effectiveness (see Fig. 1). Randomization process is centralized via a website accessible for each investigator allowing random allocation. Although patients cannot be blinded to their allocation treatment, all follow-ups assessments are gathered by assessors (psychologists) blinded to treatment allocation. Even if we cannot exclude that patients or/and caregivers inform inadvertently interviewers of the treatment they are receiving, we aim to reduce this hazard by giving explicit reminders to participants before randomization and by the use of self-measure whenever it is feasible.

Consent for participating to the study was signed by each participant after they received a full explanation in person, with the opportunity to ask questions to therapists. All participants have the right to decline participation or withdraw from the study at any time without giving a reason. The study protocol, information brochure and informed consent procedure were approved by the medical ethics committee for Human (Comité de protection des personnes d’Ile de France 1, 6th November 2017) and the study was registered in clinical trial (NCT03435705).

### Usual care

The participants allocated to the control group benefit from the usual intervention described previously [15]. Briefly, patients referred to ESA followed a standardized treatment procedure consisting of 12 to 15 home sessions performed by a therapist over a period of 3/4 months focusing on patients and potential primary caregivers. The initial assessment (1 or 2 sessions) allows the therapist to evaluate remaining abilities, and to define patients’ and caregivers’ expectations and needs. In the following sessions, patients were taught to optimize compensatory and environmental strategies to improve their performance in daily activities. Through demonstration with patients, primary caregivers were trained to use effective supervision, problem solving, manage situational distress and coping strategies to sustain patients’ social participation. Caregivers also learned to simplify activities for future decline and how to use strategies to other daily care challenges. At the end, few sessions could be devoted, according to patients and caregivers’ expectations and needs, to initiate and prepare home care support following the initial OT intervention.

### Intervention

As described above, providing meaningful and tailored activities according to patients and caregivers’ expectations and needs is the core of OT. The therapeutic approach includes environmental modifications, behavior management, physical activity, and emotional support as active components. As for the control group, the intervention group will have a first period of 3/4 months consisting of 12 to 15 home sessions; after this period the intervention group will have a maintenance of OT over 4 supplementary months with 8 extra sessions. This maintenance of OT over a longer period aims to optimize therapeutic approaches implemented during the initial 12/15 sessions by 1°) reinforcing caregivers’ confidence and competency and 2°) allowing a better matching between individual needs and further home care support.

### Outcome measures

Primary and secondary measures are assessed after the initial planned intervention (first follow-up, M4), at 8 months (second follow-up M8) and at 12 months (third follow-up, M12). All outcomes and the timetable are shown in Table 1.

**Table 1:** Overview of instruments (measures) in the MatheoAlz trial

		Inclusion	M4	M8	M12
MMSE		✓			
NPI		✓	✓	✓	✓
DAD		✓	✓	✓	✓
MADRS			✓	✓	✓
Apathy inventory			✓	✓	✓
Zarit			✓	✓	✓
RUD			✓	✓	✓
Sense of competence questionnaire			✓	✓	✓
Apathy Inventory			✓	✓	✓
QOLAD			✓	✓	✓

*MMSE*: Mini Mental State Examination

*NPI*: NeuroPsychiatric Inventory

*DAD*: Disability Assessment in Dementia

*MADRS*: Montgomery-Asberg Depression Rating Scale

*QOLAD*: Quality of Life in Dementia

*RUD*: Ressource Utilization in Dementia

#### Primary outcome measure

a) Behavior symptoms are measured with the Neuropsychiatric Inventory (NPI), that assesses the frequency and the severity of the symptoms as well as the caregiver distress in 12 behavioral domains (delusions, hallucinations, depression/dysphoria, anxiety, agitation/aggression, elation/euphoria, disinhibition, irritability/lability, apathy/indifference, aberrant motor activity, sleep/night time behavior, and appetite/eating). NPI is given as an interview questionnaire [17]. Each domain is rated by the caregiver in terms of both frequency (1–4) and severity (1–3), yielding a composite symptom domain score (frequency×severity). The total composite score is obtained by summing up the single item scores, which may range from 0 to 144, with higher scores indicating more behavioral problems.

#### Secondary outcomes

b) Quality of life is measured using the Quality of Life-Alzheimer’s disease Scale (QOL-AD) [18]. The QOL-AD covers 13 domains of quality of life. It has good internal consistency, validity and reliability and its use is recommended by the European consensus on outcome measures for psychosocial interventions in dementia [19]. The scale presents high psychometrics properties whatever the dementia etiologies [20].

c) Functional performances are measured with the Disability Assessment in Dementia (DAD). This scale assesses 10 basic and instrumental activities of daily living and decomposed each activity into initiation, organization and efficacy. The sensitivity and reliability have been established [21].

d) Apathy is measured using the apathy inventory [22], that assesses emotional fatigue, initiative loss and interest loss. Psychometric properties are good [23].

e) Depression is assessed using Montgomery-Asberg Depression Rating Scale (MADRS) [24]. This scale explores 10 dimensions: apparent sadness, expressed sadness, inner tension, reduced sleep, reduced appetite, concentration difficulties, lassitude, inability to feel, pessimistic thoughts, suicidal thoughts. The MADRS is one of the most reliable scale to detect depressive symptoms in dementia population independently of the stage of severity [25].

f) Caregivers’ burden is assessed using the Zarit Burden Index (ZBI) [26]. The ZBI is a subjective measure of burden that includes 22 items exploring the caregiver’s perception and feelings about care situations.

g) Caregivers’ sense of competence is assessed with the sense of competence questionnaire [27].

h) Vital status and institutionalization and their dates are assessed at each follow-up using any sources of data available (main caregivers or the general practitioner).

i) Patients’ resource utilization is measured for the month prior to the visit using the Resource Use in Dementia (RUD) [28]. The RUD questionnaire was specifically designed to estimate formal resource utilization (primary and secondary care consultation, hospitalizations, social care services funded or brokered by local authorities…) as well as informal care for dementia-related activities. Primary caregivers are asked to notify the number of days and time spent for formal and informal caregiving activities provided during the month prior to the visit.

j) Process of care in relation with dementia is assessed by asking caregivers for current use of anti-dementia drug, antidepressant drug, antipsychotic drug, consultation with a specialist (memory clinic, neurologist with private activity, psychiatrist, geriatrician) in the previous 3 months, speech therapist use, presence and type of home help support, financial subsidies support, and adult day care use.

### Economic evaluation

In order to provide useful and complete information for national decision makers, we propose also an economic evaluation in this project aiming at assessing:

- the total costs of patient care and disease management from a societal perspective spanning public services (hospitalization, primary and secondary care), social care services funded by local authorities as well as indirect cost associated with informal care. The extra sessions will be fully costed to generate a program cost and cost per participant;

- the cost-effectiveness ratio, expressed at the cost per avoided institutionalization at 12 months. This will be the first study to provide this kind of ratio.

- the net benefit for the French Healthcare system to widespread OT during 4 months more.

### Sample size

The sample size was based upon: a) the primary outcome (NPI) at 8 months; b) treatment effect sizes for outcome from the pilot study and other non-pharmacologic trials; c) ability to detect a clinical significant reduction of NPI score of 5 points: expected means (standard errors) were 16 (14) for control group and 11 (11) for intervention group; and d) a type I error rate of 0.05. An effect smaller than 5% would bring us at or near levels where the study could have statistical but not clinical significance as demonstrated by Mega et al. [29]. To attain 80% power for a two-sided comparison of the two treatment groups with an estimated attrition of 15%, a sample size of 240 is required.

### Statistical analyses

Analyses will be performed by intention to treat, in that all available data will be included. Methods of imputation such as last observation carried forward are of limited utility in dementia, because the expectation is decline for the usual treatment group, and most attrition will be due to death or assessment refusal. Therefore, for primary analysis missing data will be replaced by the “missing = failure” strategy, with sensitivity analyzes using maximum bias strategies, missing value modeling and nonresponse modeling, with a particular attention on death or institutionalization of patients. Depending on the outcome distribution, linear or non-linear mixed models will test the hypotheses, including the time of measurement (within-subject factor), the experimental condition (between-subject factor) and the interaction between these two factors.

## Discussion

This is an innovative randomized controlled trial that evaluates the effectiveness and cost-effectiveness of maintaining OT for people with dementia and their caregivers. This health service research is of great importance since development of OT nationwide through ESA has modified routine dementia care notably for early stages. ESAs are often the first step when the functional issues arise and constitute a cornerstone in care practice for both general practitioners and specialists’ physicians. Nevertheless, deviations to recommendations exist [15] and criticism towards the intervention format have been raised [30]. Recent study suggested that a tailored activity program delivered by occupation therapists for veterans with dementia leaded to short-term clinically relevant benefits but these benefits do not extend for a longer period after a “wash-out period” without active program [31]. Thus, it seems necessary to investigate ways to improve effectiveness of OT.

The French guidelines for dementia care offer very few evidence-based recommendations on psychosocial approaches, due to a paucity of high quality RCTs. Moreover, evidence from trials testing routine care practice is still too scarce in dementia. Because of the increase of dementia prevalence, optimizing cost-effective therapeutic approaches that primary target patients and caregivers needs appears crucial. Important advantages of the present trial nested in care settings are that the nationwide implementation could let a high level of generalizability and the tested strategy is easily replicable in routine practice.

Primary outcome measure is severity of patient’s behavioral symptoms. The latter add considerable burden to individuals and substantially contribute to health care utilization, early institutionalization and higher care costs. There are few effective and safe pharmacological treatments for behavioral symptoms leading to difficulties for addressing these troubles. As exacerbation of some symptoms often occur in the context of care provision, transfer of knowledge and specific techniques to prevent and/or manage such behaviors may be useful in daily functioning. Because the purpose of OT is to promote a sense of self, new ways to accomplish basic pleasant tasks, maintaining such therapy over a longer period may bring potential benefits on the different domains we plan to assess such as patient’s quality of life, apathy, depressive symptoms, functional impairment as well as caregiver feelings of burden and sense of competence. These outcomes are recommended for psychosocial intervention research in dementia care [19].

This study is likely to influence the availability, provisions, uptake and maintenance of OT in France and internationally, and may also impact on current evidence-based guidelines and policies relating to dementia care. Our findings will have broad clinical significance in that if positive, it would provide an evidence-based approach to help families to manage challenging behaviors. Null findings would provide a better understanding of the effect of OT’ duration on disease progression.

Implementation of cost-effective therapeutic approaches targeting early dementia stages are of great importance [32]. This study should provide evidence of the efficacy of maintenance OT on behavioral symptoms of persons with dementia and psychological symptoms in their informal caregivers.

## Abbreviations

DAD: Disability Assessment in Dementia
ESA: Equipe Spécialisée Alzheimer
MADRS: Montgomery-Asberg Depression Rating Scale
MMSE: Mini Mental State Examination
NPI: NeuroPsychiatric Inventory
QOLAD: Quality of Life in Dementia
RUD: Ressource Utilization in Dementia
